# Development of an Integrated Computational Pipeline for PARP‐1 Inhibitor Screening Using Hybrid Virtual Screening and Molecular Dynamics Simulations

**DOI:** 10.1002/open.202500021

**Published:** 2025-04-28

**Authors:** Guan Wang, Jingjing Guo, Feng Xu, Mingjuan Ji

**Affiliations:** ^1^ Taizhou Vocational and Technical College School of Medicine and Pharmaceutical Engineering Chemical Pharmaceutical Research Institute Taizhou China; ^2^ Zhejiang University of Technology College of pharmacy Hangzhou China

**Keywords:** PARP-1, Hybrid Virtual Screening, Molecular Dynamics

## Abstract

Despite the promising anticancer properties of PARP‐1 inhibitors, their clinical use is hindered by side effects. It is crucial to explore new structural variants of these inhibitors to increase efficacy and minimize side effects, enhancing their clinical viability and therapeutic scope. In this study, we developed a virtual screening workflow that synergistically integrates the capabilities of TransFoxMol, KarmaDock, and AutoDock Vina. This workflow not only streamlines the identification of potential inhibitors but also ensures a systematic approach to prioritizing candidates. Through structural clustering, we identified ten promising PARP‐1 inhibitors. Additionally, molecular dynamics simulations and MM/PBSA were employed to elucidate the binding modes of compounds 1, 3, 6, and 9 with PARP‐1, providing valuable insights into their interaction mechanisms and supporting future drug development efforts. This workflow serves as a versatile tool for early‐stage drug discovery, offering a strategic foundation for the rational design of new PARP‐1 inhibitors.

## Graphical abstract

Poly(ADP‐ribose) polymerase 1 (PARP‐1) is vital for DNA repair. A computational workflow integrating AI‐driven virtual screening (TransFoxMol, KarmaDock, AutoDock Vina) and molecular dynamics identified novel PARP‐1 inhibitors with promising efficacy and minimized side effects. Our approach offers systematic insights for advancing precision oncology drug discovery and clinical research

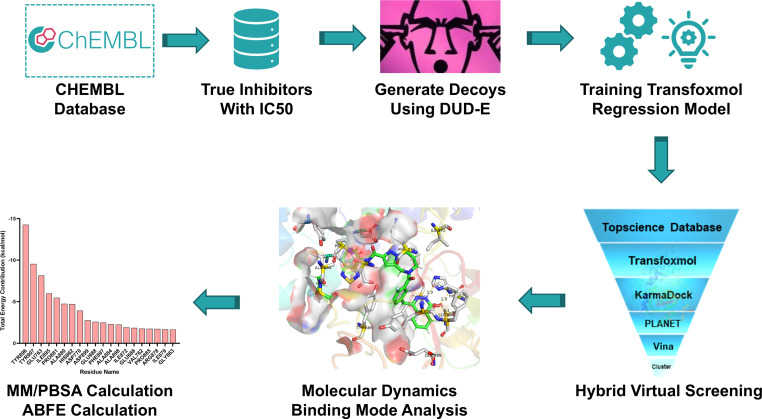

## 1 Introduction

**Figure 1:**
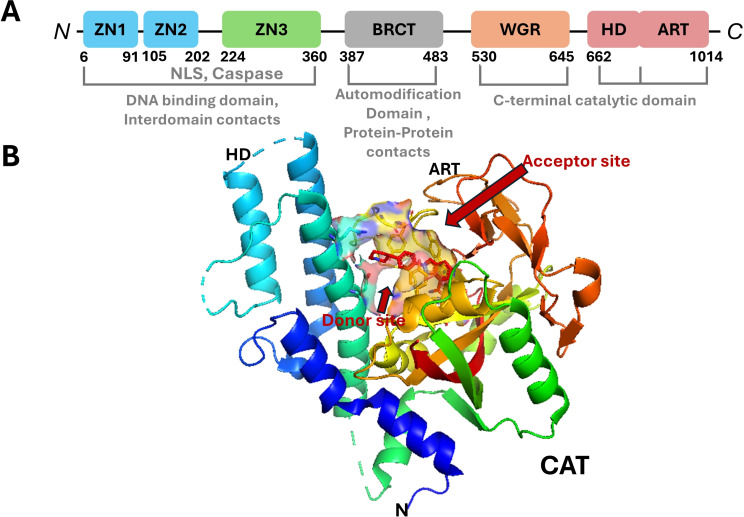
Structure of PARP‐1. (A) Schematic representation of human PARP‐1 domains. (B) The crystal structure of the catalytic domain (PDB code 7KK5^[6]^).

Poly(ADP‐ribose) polymerase 1 (PARP‐1) is a versatile enzyme pivotal in post‐translational modifications, playing a key role in several biological functions, such as DNA repair.^[1]^ PARP‐1 is a prominent member of the PARP family, which encompasses 17 distinct members.^[2]^ As depicted in Figure 1A, the structure of PARP‐1 is comprising multiple domains: the *N*‐terminal zinc finger domains (Zn1, Zn2, and Zn3), the BRCT domain, the WGR domain, and the *C*‐terminal catalytic (CAT) domain.^[3]^ The zinc finger domains Zn1 and Zn2, located at the *N*‐terminus, are specialized in recognizing specific DNA structures. The BRCT domain primarily facilitates auto‐modification.^[4]^ The CAT domain, which includes the HD and ART subdomains, is crucial for the enzyme‘s activity, mediating the addition of ADP‐ribose polymers to target proteins, thereby enhancing the DNA repair mechanism as illustrated in Figure 1B.^[5]^

**Figure 2:**
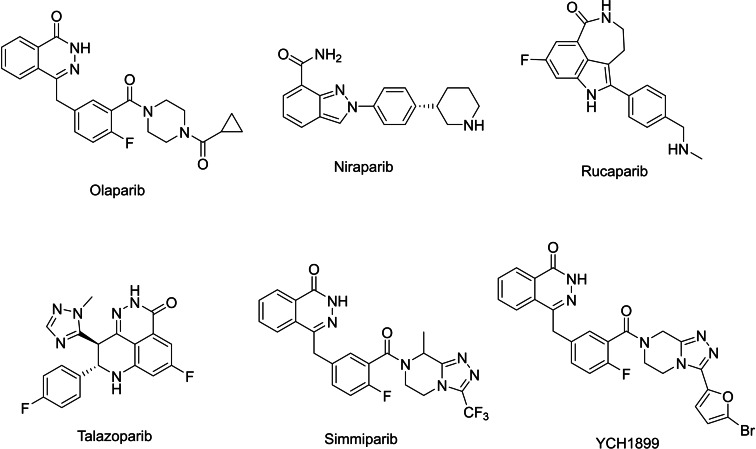
The structure of reported PARP‐1 inhibitors

During cell growth, DNA damage inevitably occurs due to internal and external factors.^[7]^ Such damage can lead to cell cycle arrest or genomic instability, both of which are key characteristics that contribute to cellular transformation into cancer. PARP‐1 inhibitors, a class of small molecule drugs targeting PARP‐1, act through two primary mechanisms: synthetic lethality and immune activation. Synthetic lethality exploits DNA repair deficiencies in tumor cells. For instance, certain cancer cells, such as those with BRCA mutations found in breast and ovarian cancers, lack the homologous recombination repair (HRR) pathway, which is necessary for repairing double‐strand breaks (DSBs). While normal cells repair DSBs through HRR, cancer cells are unable to do so.[^8^, ^9^] As a result, PARP‐1 inhibitors block the repair of single‐strand breaks (SSBs), causing these breaks to evolve into DSBs during DNA replication, leading to the death of tumor cells while sparing normal cells. On the other hand, immune activation by PARP‐1 inhibitors induces DNA damage that results in tumor cells producing more mutations and neoantigens, allowing them to be recognized and attacked by the immune system, thus achieving anticancer effects.^[10]^ The first‐generation PARP‐1 inhibitors, based on the nicotinamide structure, incorporated electron‐donating groups or bioisosteres to develop analogs like 3‐aminobenzamide (3‐AB), enhancing intermolecular interactions and inhibition efficacy. Second‐generation PARP‐1 inhibitors have improved upon drug design and structural changes to enhance potency and therapeutic outcomes. As shown in Figure 2, since the market launch of the first PARP‐1 inhibitor, Olaparib, in 2014, there have been significant breakthroughs in the development of PARP‐1 inhibitors. Subsequently, the FDA has approved several others, including Niraparib, Rucaparib, and Talazoparib, highlighting the critical role of PARP‐1 inhibitors in cancer treatment. Currently, numerous PARP‐1 inhibitors are undergoing clinical trials. For instance, Simmiparib, developed by the Shanghai Institute of Pharmaceutical Research, substitutes the triazine ring in the structure of Olaparib with a triazole ring, achieving an inhibition rate five times higher than Olaparib with an IC_50_ of 0.74 nM.^[11]^ Sun et al. have synthesized derivatives containing phthalazin‐1(2H)‐one, where YCH1899 showed significant antiproliferative activity against Olaparib and Talazoparib‐resistant cells with IC_50_ values of 0.89 and 1.13 nM, respectively.^[12]^ Despite the promising anticancer activity of PARP‐1 inhibitors, they still exhibit side effects that limit their clinical applications. Therefore, it remains essential to explore new structural types of PARP‐1 inhibitors to enhance efficacy and reduce side effects, thereby improving their clinical applicability and expanding their therapeutic potential.

In conventional computationally assisted drug design (CADD), the screening framework depends on the structures of proteins or ligands, with two main types: ligand‐based (LBVS) and structure‐based (SBVS) screening. LBVS concentrates on the structure of ligands but may neglect critical details of the target proteins, which can result in suboptimal binding effectiveness. On the other hand, SBVS uses protein structures but is often hampered by their limited availability and the difficulties in managing large‐scale databases, which can lead to increased time and computational demands. Additionally, CADD faces challenges in precisely predicting how compounds will interact with target proteins. To overcome these limitations, researchers are increasingly incorporating artificial intelligence (AI) into CADD processes to boost both the accuracy and efficiency of predictions. AI technologies, especially deep learning and attention mechanisms, are adept at capturing and processing complex molecular features and interactions. This enhances the identification of active molecules and optimizes the screening workflow. For example, the Deep Docking platform employs iterative methods to improve the precision of predictions and increase the overall efficiency of the screening process, showcasing how AI can significantly expedite the drug discovery process by refining and accelerating CADD operations.

In this study, we used hybrid virtual screening workflow to identified novel scaffold PARP‐1 inhibitor molecules. The process involves multiple stages, including for TransFoxMol, KarmaDock, and Vina for generated conformation. We also applied MM/PBSA to evaluate further binding potential between candidate compounds and PARP‐1. After several iterations of the workflow, our approach achieved promising results, with 10 compounds standing out. Furthermore, we selected three candidate molecules and performed a detailed study of the binding interactions between the compounds and their targets using molecular dynamics simulations. Therefore, we plan to conduct further molecular structure modification and optimization in the laboratory.

## 2 Computational Details and Methods

### 2.1 Structure and Database Preparation

We focused on the PARP‐1_Human catalytic domain (UniProt ID: P09874, residues 662–1011)^[13]^ and obtained 55 X‐ray co‐crystal structures from the RCSB Protein Data Bank.^[14]^ These structures underwent validation using SAVES v6.0 (https://saves.mbi.ucla.edu/, as of January 13, 2024), incorporating PROCHECK[^15^, ^16^] and ERRAT^[17]^ modules to ensure structural integrity. For virtual screening, the 7KK5 structure was prepared by removing water molecules and adding hydrogen atoms using PyMOL.

Our virtual screening workflow targeted the Topscience database (~13 million molecules, https://www.tsbiochem.com/). Using RDKit,^[18]^ we performed data preprocessing, including the removal of duplicates, parsing SMILES strings into molecular structures, filtering out invalid entries, removing salts, neutralizing charges, and verifying boron valences. Standardized SMILES formats were then generated to ensure consistency in subsequent analyses.

### 2.2 Evaluation of Docking Software

We initially downloaded active molecules from the ChEMBL database using the Uniprot ID for PARP‐1. These molecules were filtered to construct a high‐quality dataset, retaining only those with a pIC_50_ of 6 or higher. The active molecules were then clustered into 50 categories, and corresponding decoy molecules were generated using DeepCoy^[19]^ to create a dataset of inactive compounds. To screen the resulting dataset of molecules, we employed five docking software programs—KarmaDock, AutoDock‐GPU, LeDock, AutoDock Vina, and PLANET—with the EvaluationMaster^[20]^ tool for assessment. The docking results were evaluated using ROC‐AUC metrics and box plots.

We selected KarmaDock and AutoDock Vina for this study based on specific criteria. KarmaDock was chosen for its efficient handling of ligand flexibility and scoring accuracy, while AutoDock Vina was preferred due to its balance between speed and reliability in docking studies. While AutoDock‐GPU offers enhanced computational efficiency, it was not selected due to its limited flexibility in certain docking scenarios. LeDock was considered but did not align optimally with our study's computational pipeline. PLANET was not selected as it does not support conformation generation, which was essential for our analysis. These justifications for tool selection have been added to the revised manuscript to enhance transparency and clarity.

### 2.3 Virtual Screening

#### 2.3.1 TransFoxMol

TransFoxMol (https://github.com/gaojianl/TransFoxMol) combines a graph neural network with a Transformer, using chemical maps to refine attention mechanisms.^[21]^ It was trained on a curated ChEMBL dataset (as of January 12, 2024), converting IC_50_ to pIC_50_ for precision. The PARP‐1 dataset was prepared for regression using the molnetdata.py script, with hyperparameters optimized via run.py search. Key training settings included a batch size of 32, 50 epochs, a learning rate of 0.0005, and three‐fold validation. The model architecture featured eight attention heads, two layers, an output dimension of 128, and incorporated four distance thresholds (−‐D 4). Performance was evaluated by RMSE, achieving a best validation RMSE of 0.7569 and a test RMSE of 0.8109. The trained model was then applied to the Topscience database for predictions.

#### 2.3.2 KarmaDock

We utilized KarmaDock, a cutting‐edge deep learning framework designed for ligand docking, which is publicly available on GitHub (https://github.com/schrojunzhang/KarmaDock).^[22]^ This method encapsulates functionalities for expedited docking, generation and refinement of binding poses, and evaluation of binding affinities. KarmaDock's architecture is structured into a three‐stage model: (1) It employs encoders to capture the protein and ligand‘s intramolecular interaction features. (2) It utilizes E(n) equivariant graph neural networks complemented with self‐attention mechanisms to refine the ligand‘s pose, taking into account both protein‐ligand and intraligand interactions. This stage is augmented by a post‐processing step to guarantee the chemical validity of the generated structures. (3) It incorporates a mixture density network to quantitatively assess the binding affinity. In our experimental setup, the ligand was represented in ′.smi′ format for input purposes. The target protein, identified by PDB ID: 7KK5, along with its crystal‐bound ligand, was retrieved from the RCSB Protein Data Bank. Docking simulations were executed utilizing the virtual_screening_pipeline.py script.

#### 2.3.3 AutoDock Vina

AutoDock Vina (version 1.2.3)[^23^, ^24^] was deployed for ligand preparation, utilizing prepare_ligand4.py, and for generating the configuration file through prepare_pdb_split_alt_confs.py, which led to the creation of configure.txt. This file specified parameters such as: center_x=‐36.91, center_y=5.89, center_z=‐7.45; size_x=30, size_y=30, size_z=30; energy_range=3; exhaustiveness=48; num_modes=20. Following this, prepare_receptor4.py was applied for receptor preparation, which included the removal of water molecules and the confirmation of hydrogen atoms′ presence. The docking procedure was subsequently executed using Vina.

#### 2.3.4 Filtered and Cluster

In the final stage of virtual screening, we used several Python libraries for data analysis, including RDKit for cheminformatics, Matplotlib^[25]^ and Pandas^[26]^ for visualization and data handling, NumPy^[27]^ for numerical operations, SciPy^[28]^ for distance calculations, and sklearn for clustering. We loaded a dataset of docking scores from KarmaDock and Vina analyses, filtered it to retain molecules with KarmaDock scores above 80 and Vina scores below −10, yielding 180 molecules for clustering. Morgan fingerprints^[29]^ were generated for each molecule to represent their structural features as fixed‐length bit vectors. Pairwise Jaccard distances^[30]^ between fingerprints quantified chemical dissimilarities, serving as input for agglomerative hierarchical clustering. Using average linkage and these distances, molecules were grouped into ten clusters, reflecting their chemical diversity.

### 2.4 Molecular Dynamics Simulation and MM/PBSA

We employed GROMACS^[31]^(version 2020.7) for molecular dynamics (MD) simulations to investigate protein‐ligand interactions. Structures were refined using pdbfixer and parameterized with the amber99sb‐ildn force field. Simulations were conducted in a 1.5 nm cubic box with the tip3p water model. Ligand structures were converted to mol2 format using Open Babel and parameterized with ACPYPE,^[32]^ while protein topology was generated using pdb2gmx and combined with ligand parameters. Energy minimization used the Conjugate Gradient method with the ‐DFLEXIBLE option, ceasing when forces fell below 1000 kJ/mol/nm. The NVT equilibration phase employed a leap‐frog integrator over 500 ps, with 1.0 ps recording intervals, using the Particle Mesh Ewald method for electrostatics and the Berendsen thermostat at 300 K. This was followed by NPT equilibration using the Berendsen method at 1.0 bar. The production run spanned 500 ns, with data recorded every 100 ps, employing the LINCS algorithm for constraining hydrogen bonds, and V‐rescale and Parrinello‐Rahman methods for temperature and pressure control. Each simulation was performed in duplicate to ensure reproducibility and robustness.

For post‐simulation analysis, we performed MM‐PBSA calculations using Jerkwin's gmxtools (https://github.com/Jerkwin/gmxtools)^[33]^ through a custom bash script. This script automated the extraction of necessary files, including trajectory (.xtc), topology (.tpr), and index files (*.ndx), streamlining the preparation process. The Adaptive Poisson‐Boltzmann Solver (APBS)^[34]^ (version 3.0.0) was utilized for calculating electrostatic potentials. The workflow enabled efficient setup and estimation of binding free energies, incorporating contributions from molecular mechanics, electrostatic interactions, and solvation energies for precise free energy calculations.

### 2.5 Absolute Binding Free Energy Perturbation Calculation using LumiNet

To enhance the scoring and binding energy evaluation of compounds identified from virtual screening, Absolute Binding Free Energy Perturbation (ABFEP) calculations were performed using LumiNet.^[35]^ This computational method quantifies the binding free energy of ligands with high accuracy by simulating molecular interactions under various perturbative conditions. LumiNet was employed to analyze the virtual screening hits, providing detailed insights into the relative binding affinities of the ligands to the target protein. The resulting binding energy data contributed to a more refined ranking of the screened compounds.

## 3 Results and Discussion

### 3.1 The Evaluation of PARP‐1 Structures

**Figure 3:**
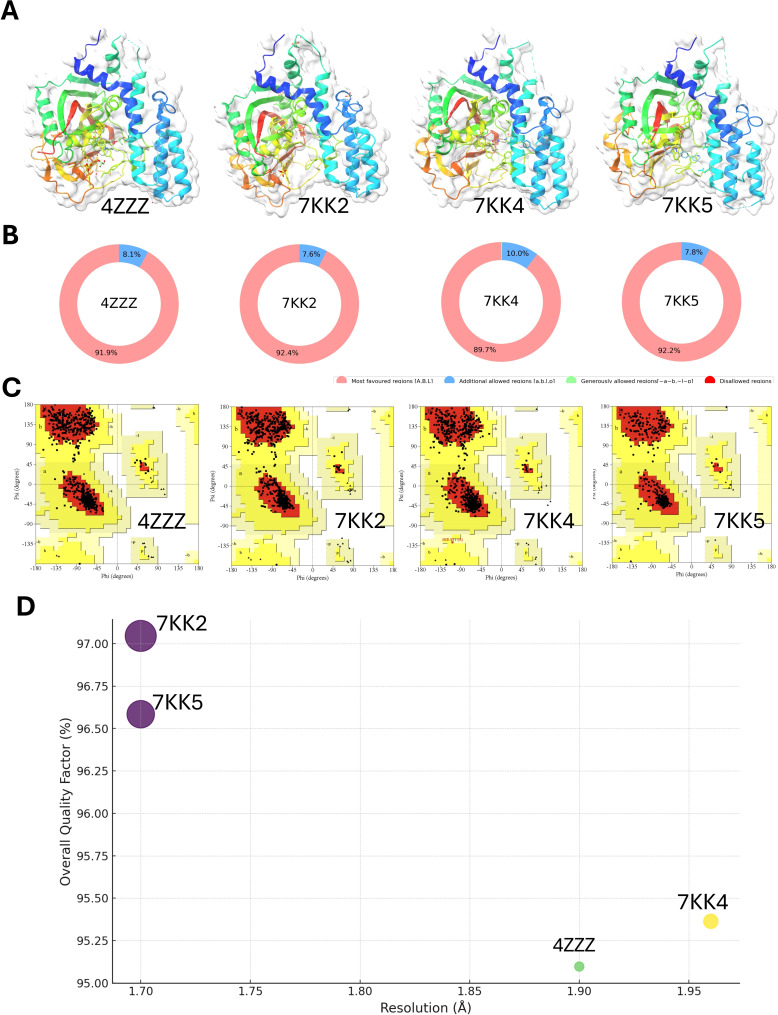
**Comprehensive Structural Analysis of Proteins**. (A) 4ZZZ X‐Ray Diffraction Structure at 1.90 Å Resolution, 7KK2 X‐Ray Diffraction Structure at 1.70 Å Resolution, 7KK4 X‐Ray Diffraction Structure at 1.96 Å Resolution, and 7KK5 X‐Ray Diffraction Structure at 1.70 Å Resolution. (B) Distribution Analysis of Amino Acid Residues in Preferred Conformational Regions for 4ZZZ, 7KK2, 7KK4, and 7KK5. (C) Ramachandran Plots Highlighting Residue Distribution of 4ZZZ, 7KK2, 7KK4, and 7KK5. (D) Quality Assessment of Protein Models via ERRAT and Resolution.

In our quest to identify the optimal PARP‐1 protein structure for our study, we initiated our search within the UniProt database for the PARP‐1 enzyme specific to Homo sapiens (Human), bearing the UniProt ID P09874 (https://www.uniprot.org, access date: 2024–1‐13), and identified 55 PARP‐1_HUMAN structures containing catalytic domain (662‐1011). Our selection criteria excluded any structures exhibiting mutations or those with a resolution exceeding 2 Å, to ensure high fidelity in our computational analyses (Figure 3A, Table 1).

**Table 1:** PARP1 structure Quality Metrics and Ramachandran Plot Statistics.

PDB ID	Mutation	Resolution	Released	Overall quality factor	VERIFY 3D	Ramachandran plot
4ZZZ	No	1.90 Å	2015‐08‐12	95.0966	Pass	91.9 % core 8.1 % allow 0.0 % gener 0.0 % disal
7KK2	No	1.70 Å	2021‐01‐06	97.0458	Pass	92.4 % core 7.6 % allow 0.0 % gener 0.0 % disa
7KK4	No	1.96 Å	2021‐01‐06	95.3632	Pass	89.8 % core 10.0 % allow 0.2 % gener 0.0 % disal
7KK5	No	1.70 Å	2021‐01‐06	96.5839	Pass	92.2 % core 7.8 % allow 0.0 % gener 0.0 % disal

Further scrutiny was directed towards four remaining structures, evaluated using the SAVES v6.0 platform, a comprehensive tool for assessing structural integrity and reliability. This analysis encompassed several aspects; firstly, VERIFY 3D inspection was applied to assess the compatibility of each structure‘s amino acid sequence with its 3D structure, with all nine structures surpassing the threshold of 80 points (Figure 3B, Table 1). Subsequent examination employed the PROCHECK module, which gauges the stereochemical quality of the protein structures. Among the structures analyzed, 7KK4 was noted for having less than 90 % of its residues in the most preferred regions, leading to its exclusion from further consideration (Figure 3C, Table 1).

Additionally, the ERRAT module, designed for the evaluation of crystallography‐derived protein structures, provided an overall quality factor for each structure (Figure 3D, Table 1). Notably, structure 7KK2 emerged as the optimal choice based on this metric; however, it was ultimately deemed unsuitable due to the absence of small molecule compounds within its structure. Considering both resolution and PROCHECK scores, we concluded that structure 7KK5 represented the best candidate for the foundation of our virtual screening efforts in this study.

### 3.2 The Evaluation of Docking Software

#### 3.2.1 Docking Model Evaluation

**Figure 4:**
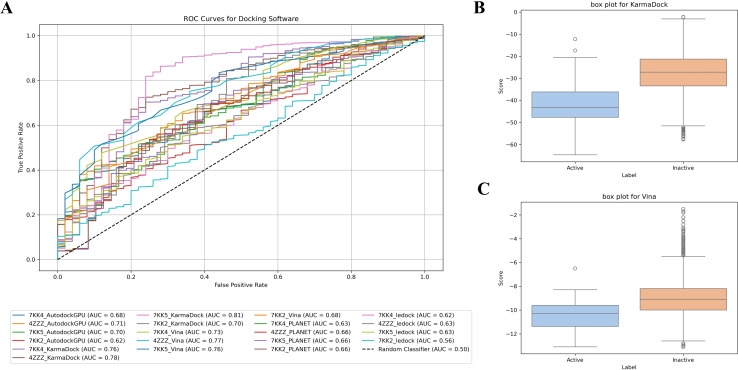
Evaluation result of 5 docking softwares for PARP1. (A) The ROC curves analysis for docking software. (B) The box plot of KarmaDock. (C) The box plot of Vina.

Based on the results of protein structure assessments, we evaluated five docking software programs: KarmaDock, Autodock‐GPU, LeDock, AutoDock Vina, and PLANET. The evaluation results are presented in Figure 4. Our analysis reveals that KarmaDock demonstrates the best overall performance among all evaluated docking software, followed by Vina, Autodock‐GPU, and PLANET, with LeDock showing the weakest performance. The box plot indicates that KarmaDock effectively distinguishes between active and inactive compounds (Figure 4B–C). ROC‐AUC analysis further supports that the protein with RCSB ID 7KK5 paired with KarmaDock achieves the highest ROC‐AUC of 0.81, while the protein with RCSB ID 4ZZZ paired with AutoDock Vina achieves a ROC‐AUC of 0.77. However, since KarmaDock does not reliably predict the 3D poses after docking, we ultimately selected KarmaDock and Vina for the virtual screening process, along with the protein 7KK5, which demonstrated the best evaluation results, to ensure the identification of high‐quality potential active compounds and generate accurate docking poses that provide reliable structural information for subsequent molecular dynamics simulations.

#### 3.2.2 Redocking of Co‐Crystallized Ligand

**Figure 5:**
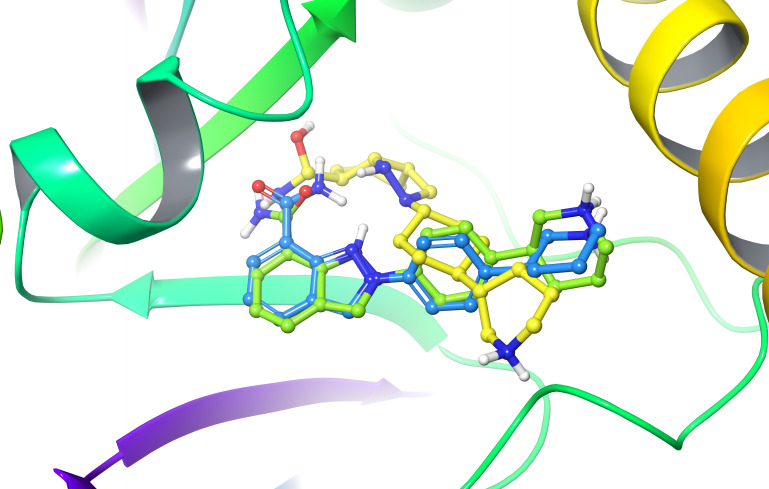
Comparison of the original structure of PARP‐1 (blue, PDB ID: 7KK5) and the conformation after redocking using KarmaDock(yellow) and AutoDock Vina(green)

To further validate the accuracy and reliability of our screening protocol, we performed redocking of the co‐crystallized ligand and calculated the root mean square deviation (RMSD) between the structures before and after redocking. Structurally, the conformation of the co‐crystallized ligand after redocking is essentially identical to its original conformation (Figure 5). The RMSD calculated using Open Babel was 1.650 Å (KarmaDock) and 1.032 Å (Vina), indicating that the conformations of the co‐crystallized ligand before and after redocking are highly similar. Such minor differences typically do not significantly affect biological function or ligand binding.

### 3.3 Hybrid Virtual Screening

**Figure 6:**
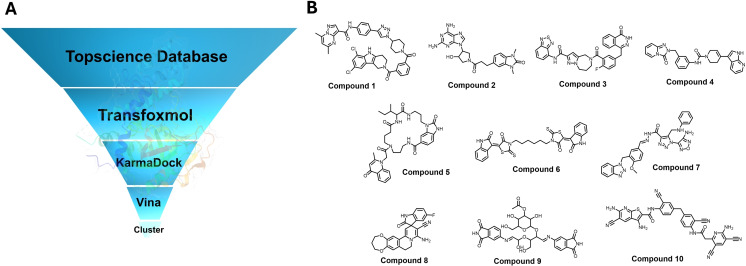
The Hybrid Virtual screening workflow. (A) Overview of the virtual screening process targeting PARP‐1. (B) Structural representation of the final selected 10 small molecules.

In our pursuit to identify potent PARP‐1 inhibitors, we adopted an integrative approach, combining AI‐driven screening methodologies with conventional computer‐aided drug design (CADD) tools (Figure 6A). Initially, we leveraged TransFoxMol, a cutting‐edge tool recognized for its innovative integration of multi‐scale 2D molecular environments within a graph neural network and Transformer architecture framework. TransFoxMol excels in correlating molecular structures with their properties, thereby facilitating an efficient initial screening of the PAPR1 activity dataset sourced from the ChEMBL database. Through this initial phase, we utilized TransFoxMol to screen the extensive Topscience database (total of 13 million molecules), filtering for molecules predicted with a score above 7.5, resulting in 189,480 candidates.

For the subsequent round of screening, we employed KarmaDock, an AI‐based docking tool renowned for its precision in rapidly predicting the binding strength of protein‐ligands. By setting a threshold Karma score of above 80, we further refined our selection to 3,471 molecules. Despite the strengths of AI in predicting molecular activity and affinity, these tools often fall short in accurately generating 3D conformations of compounds. To address this limitation, we incorporated AutoDock Vina, a traditional molecular docking tool, to enhance the precision of compound conformations. Focusing on molecules that achieved a KarmaDock score above 80 and a AutoDock Vina score below −10 kcal/mol, we distilled our list to 180 compounds.

To assess the structural diversity within our dataset, we utilized Morgan molecular fingerprints, a method allowing for the detailed categorization of molecules into one of ten pre‐established clusters reflecting their chemical similarity. This clustering enabled us to distill our findings further to ten unique molecules (Figure 6B and Table 2). The selection criterion for further analysis was primarily based on achieving the highest Vina docking scores, from which compounds 1, 3, 6, and 9 were chosen for advanced Molecular Dynamics (MD) simulations. Then We performed Absolute Binding Free Energy Perturbation (ABFEP) calculations on the top 10 screened compounds to evaluate their binding affinities and stability with PARP‐1. All compounds exhibited binding free energy values superior to −8 kcal/mol, indicating strong binding affinities and robust interaction stability with the target protein.

**Table 2:** Each stage score of the final selected 10 small molecules.

Entry	InChIKey	TransFoxMol	KarmaDock	Vina	MM/PBSA (kcal/mol)	ABFEP (kcal/mol)
Compd. 1	InChIKey=ORBZKIMBPNECHS‐UHFFFAOYSA‐N	7.72	93.26	‐12.89	‐24.01	‐9.43
Compd. 2	InChIKey=YMJJZAYPAWMBMO‐PTTDRDKLNA‐N	7.84	82.22	‐10.80	‐	‐8.58
Compd. 3	InChIKey=ULUVBBCDTWTZLD‐UHFFFAOYSA‐N	8.54	94.83	‐14.21	‐40.08	‐9.50
Compd. 4	InChIKey=HYUHVOLQYDHZHQ‐UHFFFAOYSA‐N	7.59	85.07	‐11.48	‐	‐9.69
Compd. 5	InChIKey=DJIKKGWKXJGKRI‐WYOOIXGGSA‐N	7.50	108.38	‐10.80	‐	‐10.28
Compd. 6	InChIKey=DSYYGKZYBMLNEM‐FLFKKZLDSA‐N	7.78	88.67	‐11.57	‐20.81	‐10.57
Compd.7	InChIKey=NDIZSXKXYRQMAU‐SXGWCWSVSA‐N	7.64	81.21	‐10.84	‐	‐8.06
Compd. 8	InChIKey=ANAHWPGZGLJKPD‐UHFFFAOYNA‐N	7.59	80.59	‐10.80	‐	‐9.34
Compd. 9	InChIKey=RVNGBBQZELJWBB‐UHFFFAOYSA‐N	8.04	93.07	‐11.85	‐12.88	‐8.95
Compd.10	InChIKey=GCVOEPYFYISOOG‐UHFFFAOYSA‐N	7.60	85.93	‐11.49	‐	‐8.6

This approach, a synergy of hybrid virtual screening and structural clustering, integrates the predictive prowess of artificial intelligence with the robust methodologies of CADD technologies. By doing so, we not only streamline the identification process of promising PARP‐1 inhibitor candidates but also furnish future researchers with novel PARP‐1 scaffolds.

### 3.4 Determination of Structural Stability and Dynamical of the Complexes

The determination of structural stability and dynamics of complexes is a critical phase in the computational analysis of protein‐ligand interactions. This step involves conducting Molecular Dynamics (MD) simulations, a computational method that enables the exploration of the physical movements of atoms and molecules over time. Here, our objective is to evaluate the behavior of selected PARP‐1 inhibitor complexes under simulated physiological conditions, providing insights into their conformational stability, flexibility, and overall dynamic behavior.

The changes in interaction forces before and after molecular dynamics simulations are crucial indicators for understanding the stability of protein‐ligand complexes and their biological functions. By observing these changes, we can analyze the interactions between molecules, such as hydrogen bonds, van der Waals forces, and electrostatic interactions, to determine whether significant adjustments have occurred. These alterations in interaction forces can reveal the dynamic adjustments of molecular conformations in realistic environments, aiding in the explanation of complex stability and potential dissociation behaviors.

**Figure 7:**
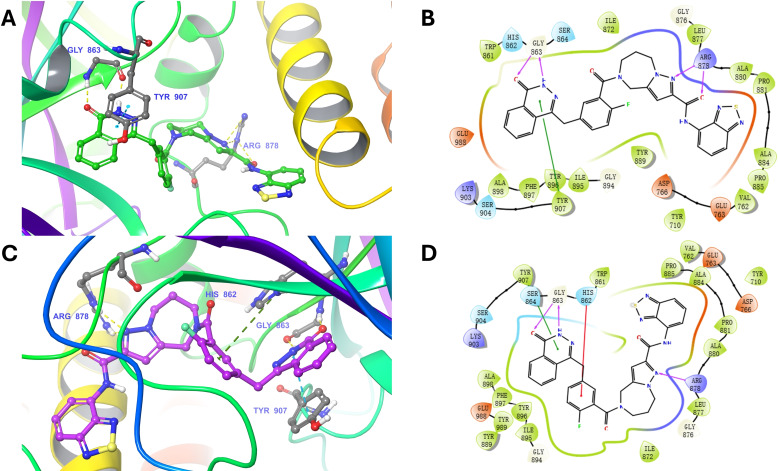
The Binding mode of PARP‐1‐compound 3 complex after docking and MD simulation. (A). Three‐dimensional visualization of the binding mode of PARP‐1‐compound 3 after docking. (B) Two ‐dimensional visualization of the binding mode of PARP‐1‐compound 3 after docking. (C) Three‐dimensional visualization of the binding mode of PARP‐1‐compound 3 after MD simulation. (D) Two‐dimensional visualization of the binding mode of PARP‐1‐compound 3 after MD simulation.

In the binding mode of compound 3 with PARP‐1(Figure 7), the key interactions include hydrogen bonds and pi‐pi stacking. Two hydrogen bonds were formed with ARG878 and GLY863, respectively, while a pi‐pi interaction was established with TYR907. Following molecular dynamics (MD) simulation, the interaction pattern shifted slightly. The hydrogen bonds with ARG878 decreased to one, whereas the two hydrogen bonds with GLY863 remained intact. The pi‐pi interaction with TYR907 was maintained, ensuring the ligand‘s stability within the binding pocket. Additionally, a new pi‐cation interaction formed with HIS862, which likely enhances the ligand's stability in the active site post‐simulation.

These modifications highlight the dynamic nature of molecular interactions observed during MD simulations, where slight changes in binding poses or environmental factors can modify the number and type of interactions. The appearance of the pi‐cation interaction with HIS862 suggests an increased affinity of compound 3 for the binding pocket, potentially improving its binding stability and overall efficacy as a ligand. This underscores the importance of analyzing post‐docking dynamics to fully understand ligand behavior.

**Figure 8:**
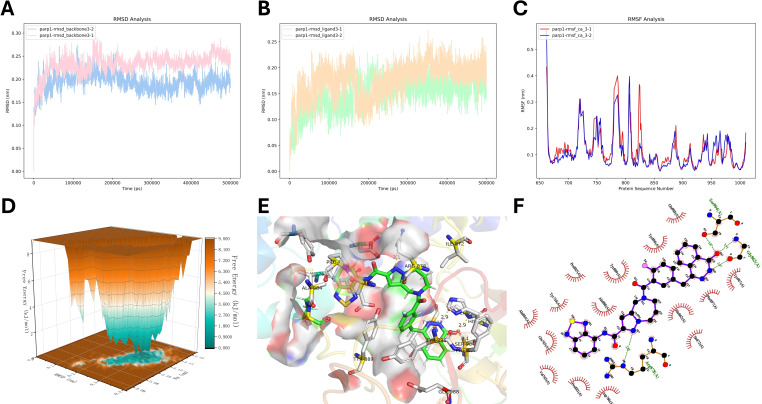
Comprehensive Analysis of compound 3 and PARP‐1 Interactions. (A) RMSD values for PARP‐1. (B) RMSD values for compound 3. (C) RMSF of the C‐alpha of compound 3. (D) Gibbs Free Energy landscape of compound 3/PARP‐1. (E) Three‐dimensional visualization of the PARP‐1‐compound 3 interaction. (F) Two‐dimensional representation of the PARP‐1‐compound 3 interaction.

As shown in Figures 8A–B, the backbone and ligand trajectories of the PARP‐1‐compound 3 complex reached equilibrium during the last 10 ns of molecular dynamics (MD) simulation. Figure 8C presented the root mean square fluctuation (RMSF) of PARP‐1, revealing fluctuations in amino acid residues ranging from 0.1 Å to 0.6 Å. Additionally, Gibbs free energy landscape analysis (Figures 8D, S4D, S5D, and S6D) identified the most energetically favorable compounds as 1, 3, 6, and 9, with compound 3 showing the optimal free energy profile. Compound 3 formed one hydrogen bond with ARG878, two hydrogen bonds with GLY863, a π‐π interaction with TYR907, and a pi‐cation interaction with HIS862. These interactions align with the known key residues within the PARP‐1 binding pocket, particularly HIS862, GLY863, and TYR907, which are reported to play critical roles in ligand binding.

These combined interactions suggest that compound 3 exhibits strong stability and binding specificity, making it a viable candidate for further development as a novel PARP‐1 inhibitor. Its robust interaction with critical residues in the PARP‐1 active site enhances its potential therapeutic effectiveness, particularly in the context of targeting DNA repair pathways in cancer therapy.

As described in figure S1 and S4, compound 1 formed a consistent pi‐cation interaction with Arg878 in the PARP‐1 binding pocket before and after molecular dynamics (MD) simulation, indicating that this residue may be a key amino acid in the active site. Although compound 1 has relatively few interactions with PARP‐1, its structure effectively occupies the binding pocket cavity, contributing to a certain degree of binding stability. Additionally, In the case of compound 6(Figure S2 and S5), hydrogen bonding with ARG865 and pi‐pi interaction with TYR907 were observed during docking; however, after the MD simulation, the hydrogen bond with ARG865 disappeared, and a new pi‐cation interaction formed with GLU988. In contrast, compound 9 consistently formed hydrogen bonds with HIS862, GLY863, and MET890 both before and after the simulation, and engages in pi‐pi interactions with TYR889 and TYR907(Figure S3 and S6). These interactions suggest that compound 9 has strong binding stability with PARP‐1, highlighting its potential for further development as a PARP‐1 inhibitor.

### 3.5 Binding Free Energy Calculations

In the realm of drug discovery, accurately assessing the binding free energy is essential for evaluating the efficacy and specificity of drug candidates. The Molecular Mechanics/Poisson‐Boltzmann Surface Area (MM/PBSA) method stands out as a crucial technique in this process due to its optimal balance between computational intensity and accuracy. In our study, MM/PBSA analyses were performed using data from molecular dynamics (MD) simulation trajectories. These analyses shed light on the contributions of individual amino acid residues to the overall binding free energy in various PARP‐1/compound complexes. As depicted in Figure 6, negative values indicate beneficial interactions that enhance the stability of the complex. For instance, the binding free energy of compound 1 with PARP‐1 was calculated to be −24.013 kcal/mol, with significant contributions from residues like PRO881, TYR907, and TYR889 (Figure SB, Table 2). Similarly, compound 3 demonstrated a binding free energy of −40.081 kcal/mol with notable contributions from TYR896, TYR907, and GLU763 (Figure 8B, Table 2). Compound 6 showed a binding free energy of −20.806 kcal/mol, influenced significantly by TYR896, TYR907, and GLU889 (Figure SB, Table 2). Lastly, a repeat analysis for compound 6/PARP‐1 presented a binding free energy of −12.880 kcal/mol with contributions from TYR907, TYR896, and ASN868 being particularly impactful (Figure SB, Table 2).

### 3.6 Occupancy and Distances Calculation

**Figure 9:**
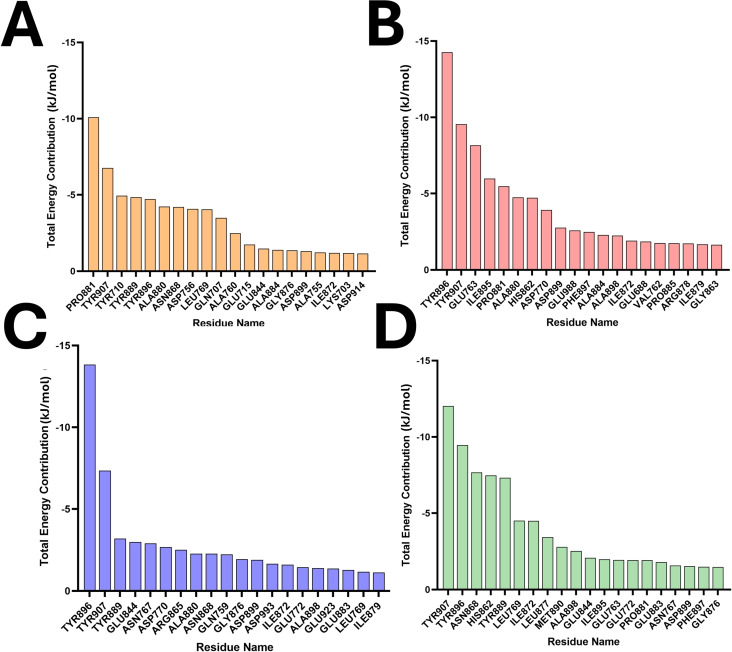
The major contributions of individual amino acid residues to the interaction complex of PARP1 with compounds (A) 1, (B) 3, (C) 6, (D) 9.

**Figure 10:**
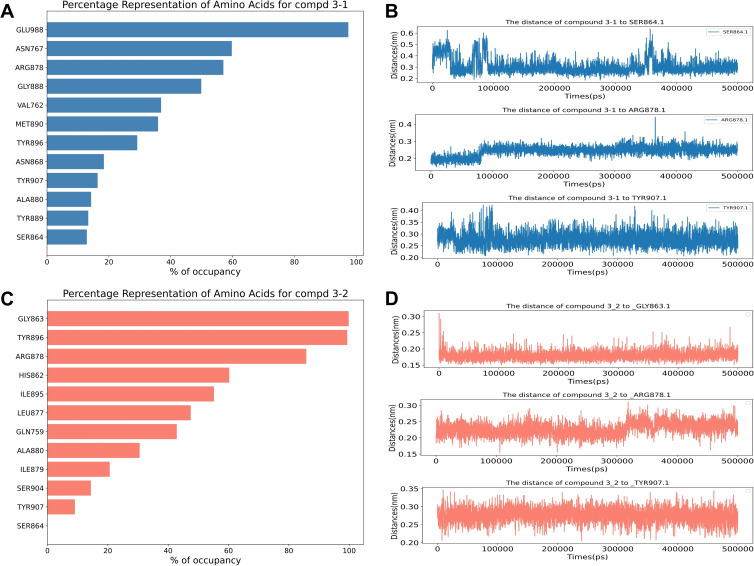
The occupancy (A) and the binding distances (B) between compound 3–1 and different residues of PARP‐1 protein, and the occupancy (C) and the binding distances (D) between compound 3–2 and different residues of PARP‐1 protein.

The analysis of the contact frequency and distance between the protein pocket residues and the ligand throughout the simulation can help confirm the binding mechanism and guide subsequent modifications based on key amino acids identified as crucial(Figure 9). In our study, we selected compound 3 (Figure 9B), which exhibited the best performance, and conducted a more in‐depth analysis of amino acid contacts and distances based on two independent molecular dynamics simulations. As shown in Figures 10A and 10C, the occupancy of ARG878 consistently exceeded 50 %, and its distance from compound 3 remained below 3 Å (Figures 10B, D), supporting the formation of a hydrogen bond between them. Additionally, in the second replication experiment, GLY863 exhibited nearly 100 % occupancy (Figure 10C), and its distance from compound 3 remained stable at around 2.5 Å (Figure 10D), suggesting the formation of a strong hydrogen bond. However, in the first replication experiment, GLY863′s occupancy was not observed, indicating that the interaction between compound 3 and GLY863 may not be stable. This suggests a potential direction for optimization to strengthen the hydrogen bond with GLY863 and enhance the stability of the compound 3‐PARP‐1 complex. Moreover, TYR907 and HIS862 also exhibited certain levels of occupancy, possibly forming π‐π stacking and hydrophobic interactions, which are also reflected in the residue energy contributions shown in Figure 9B.

## 4 Conclusions

Despite the promising anticancer activity of PARP‐1 inhibitors, their side effects often limit clinical application. Thus, developing new structural types of PARP‐1 inhibitors with enhanced efficacy and reduced side effects is crucial for improving their clinical viability and expanding their therapeutic potential. In this study, we established a robust screening workflow to support the discovery and design of novel PARP‐1 inhibitors. Our methodology integrated TransFoxMol, KarmaDock, and AutoDock Vina, leveraging these tools for rescoring and conformational generation. This combined approach facilitated the identification of structurally diverse candidates from the Topscience database, providing valuable insights into the structural characteristics that contribute to potent PARP‐1 inhibition.

This workflow contributes significantly to clinical research and drug development by enabling a more systematic and efficient identification of potential PARP‐1 inhibitors with improved profiles. It offers a deeper mechanistic understanding of protein‐ligand interactions, which can guide the rational design of next‐generation inhibitors with minimized side effects. The insights gained from this study can accelerate preclinical development, streamline candidate selection, and potentially lead to the development of more effective and safer therapies, ultimately improving clinical outcomes for patients.

## Conflict of Interests

There are no conflicts of interest to declare.

## Supporting information

As a service to our authors and readers, this journal provides supporting information supplied by the authors. Such materials are peer reviewed and may be re‐organized for online delivery, but are not copy‐edited or typeset. Technical support issues arising from supporting information (other than missing files) should be addressed to the authors.

Supporting Information

## Data Availability

If you need research data related to the article, please request it from the corresponding author.
