# Supplementary material for: Development of an Integrated Computational Pipeline for PARP‐1 Inhibitor Screening Using Hybrid Virtual Screening and Molecular Dynamics Simulations
Source: ChemistryOpen. 2025 Apr 28;14(8):e202500021. doi: 10.1002/open.202500021 (PMC12368897; doi:10.1002/open.202500021)
Supplement: Supplementary file 1 — Supporting Information [file OPEN-14-e202500021-s001.pdf]

ChemistryOpen

Supporting Information

Development of an Integrated Computational Pipeline for PARP-1 Inhibitor Screening Using Hybrid Virtual Screening and Molecular Dynamics Simulations

Guan Wang,* Jingjing Guo, Feng Xu, and Mingjuan Ji

Supporting Information

Development of an Integrated Computational Pipeline for PARP-1 Inhibitor Screening Using Hybrid Virtual Screening and Molecular Dynamics Simulations

Authors:

Guan Wang [a]*, Jingjing Guo[a], Feng Xu[a], Mingjuan Ji [b]

a. Taizhou Vocational and Technical College, School of Medicine and Pharmaceutical Engineering, Chemical Pharmaceutical Research Institute, Taizhou, China; guanwang@tzvtc.edu.cn; guojingjing@tzvtc.edu.cn; xufeng901@tzvtc.edu.cn

b. Zhejiang University of Technology, College of pharmacy, Hangzhou, China; 221122070215@zjut.edu.cn

* Correspondence: Guan Wang, guanwang@tzvtc.edu.cn

1. Supplementary Figures and Tables

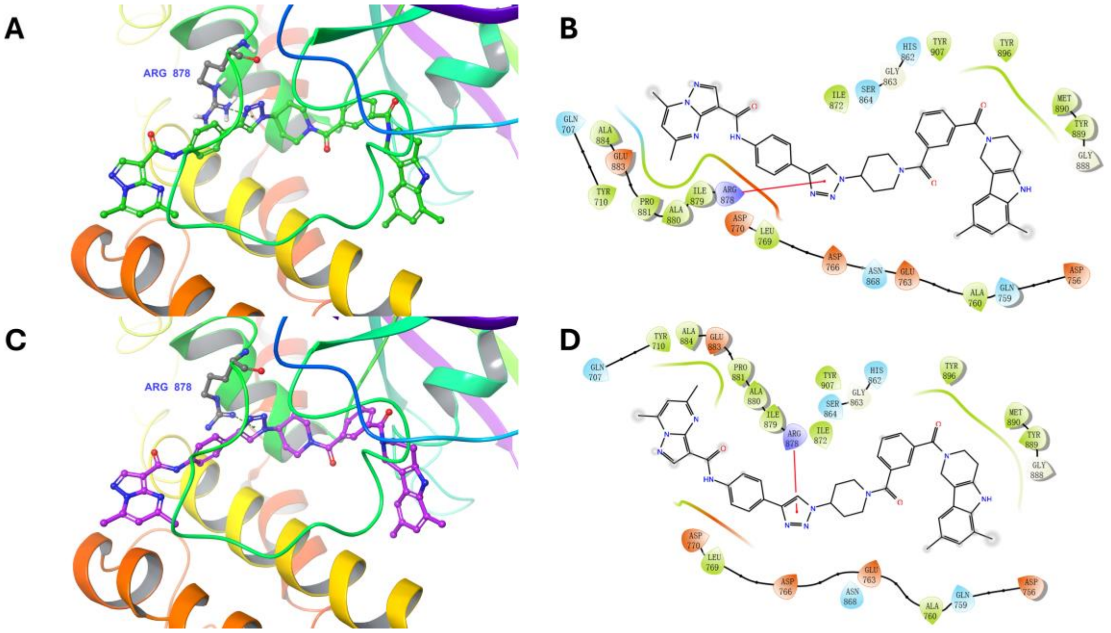

Figure S1. The Binding mode of PARP-1-compound 1 complex after docking and MD simulation. (A). Three-dimensional visualization of the binding mode of PARP-1-compound 1 after docking. (B) Two -dimensional visualization of the binding mode of PARP-1-compound 1 after docking. (C) Three-dimensional visualization of the binding mode of PARP-1-compound 1 after MD simulation. (D) Two-dimensional visualization of the binding mode of PARP-1-compound 1 after MD simulation.

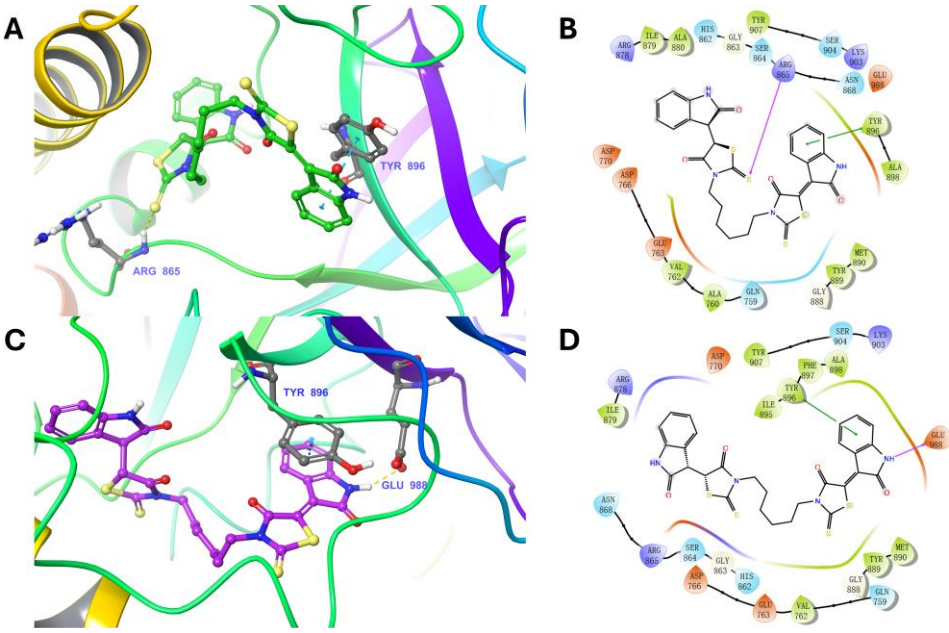

Figure S2. The Binding mode of PARP-1-compound 6 complex after docking and MD simulation. (A). Three-dimensional visualization of the binding mode of PARP-1-compound 6 after docking. (B) Two -dimensional visualization of the binding mode of PARP-1-compound 6 after docking. (C) Three-dimensional visualization of the binding mode of PARP-1-compound 6 after MD simulation. (D) Two-dimensional visualization of the binding mode of PARP-1-compound 6 after MD simulation.

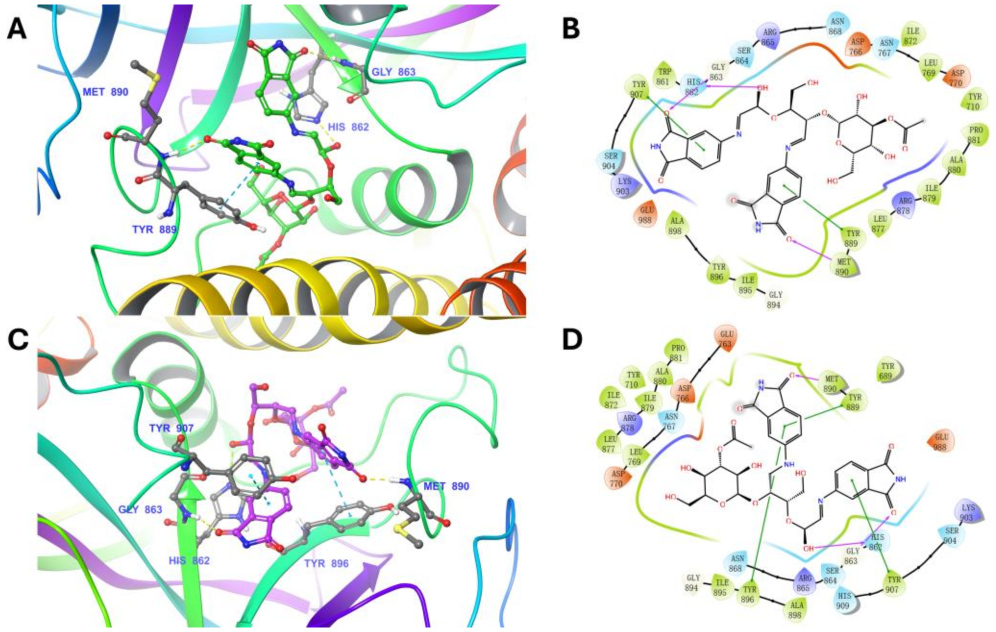

Figure S3. The Binding mode of PARP-1-compound 9 complex after docking and MD simulation. (A). Three-dimensional visualization of the binding mode of PARP-1-compound 3 after docking. (B) Two -dimensional visualization of the binding mode of PARP-1-compound 3 after docking. (C) Three-dimensional visualization of the binding mode of PARP-1-compound 3 after MD simulation. (D) Two-dimensional visualization of the binding mode of PARP-1-compound 3 after MD simulation.

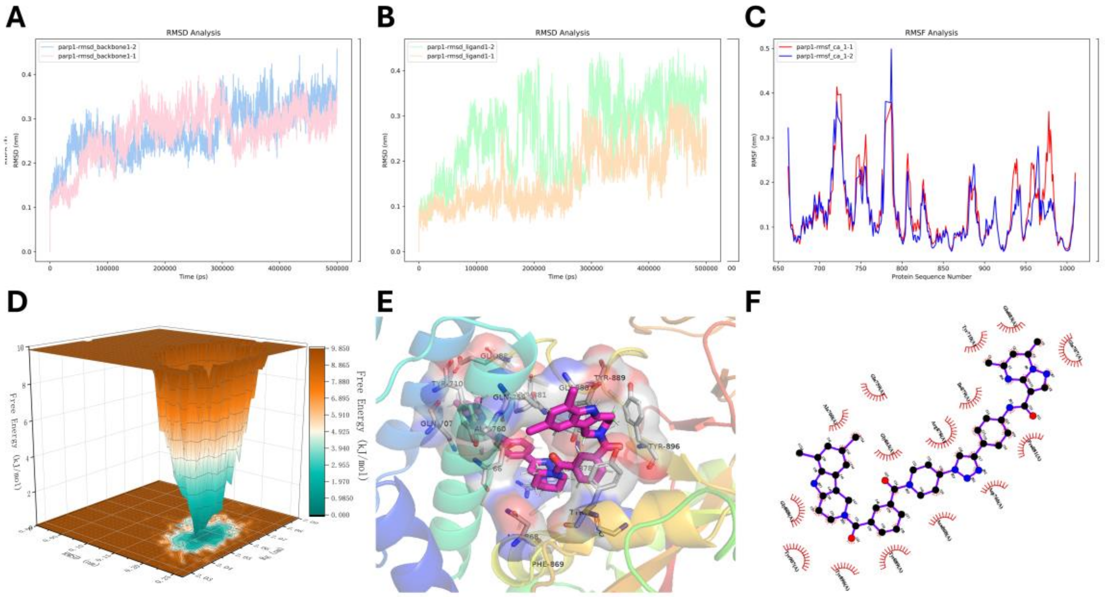

Figure S4. Comprehensive Analysis of compound 1 and PARP-1 Interactions. (A) RMSD values for PARP-1. (B) RMSD values for compound 1. (C) RMSF of the C-alpha of compound 1. (D) Gibbs Free Energy landscape of compound 1/PARP-1. (E) Three-dimensional visualization of the PARP-1-compound 1 interaction. (F) Two-dimensional representation of the PARP-1-compound 1 interaction.

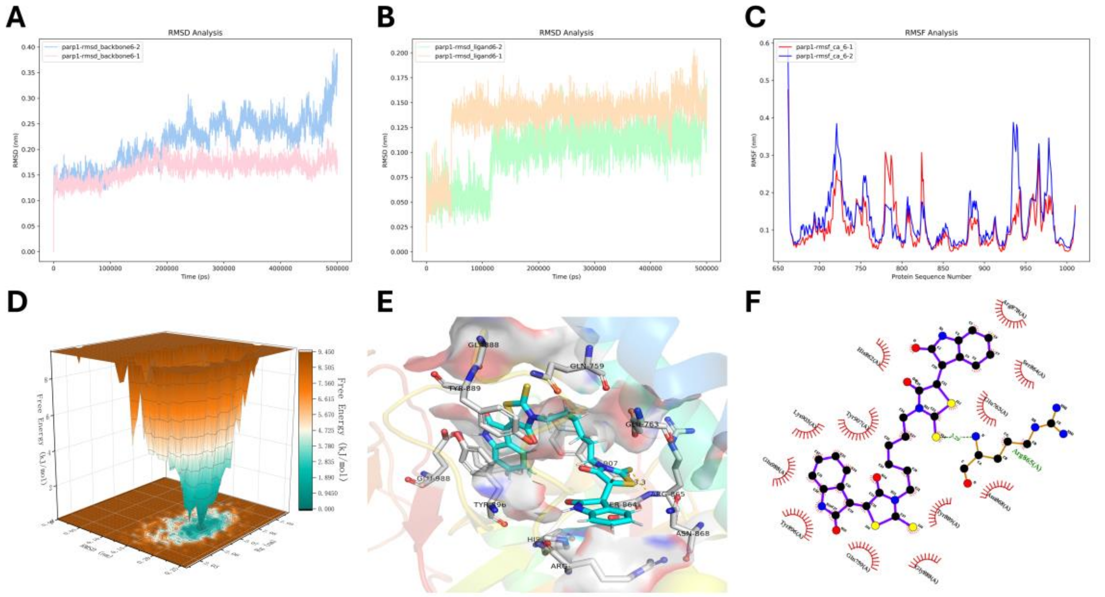

Figure S5. Comprehensive Analysis of compound 6 and PARP-1 Interactions. (A) RMSD values for PARP-1. (B) RMSD values for compound 6. (C) RMSF of the C-alpha of compound 6. (D) Gibbs Free Energy landscape of

compound 6/PARP-1. (E) Three-dimensional visualization of the PARP-1-compound 6 interaction. (F) Two-dimensional representation of the PARP-1-compound 6 interaction.

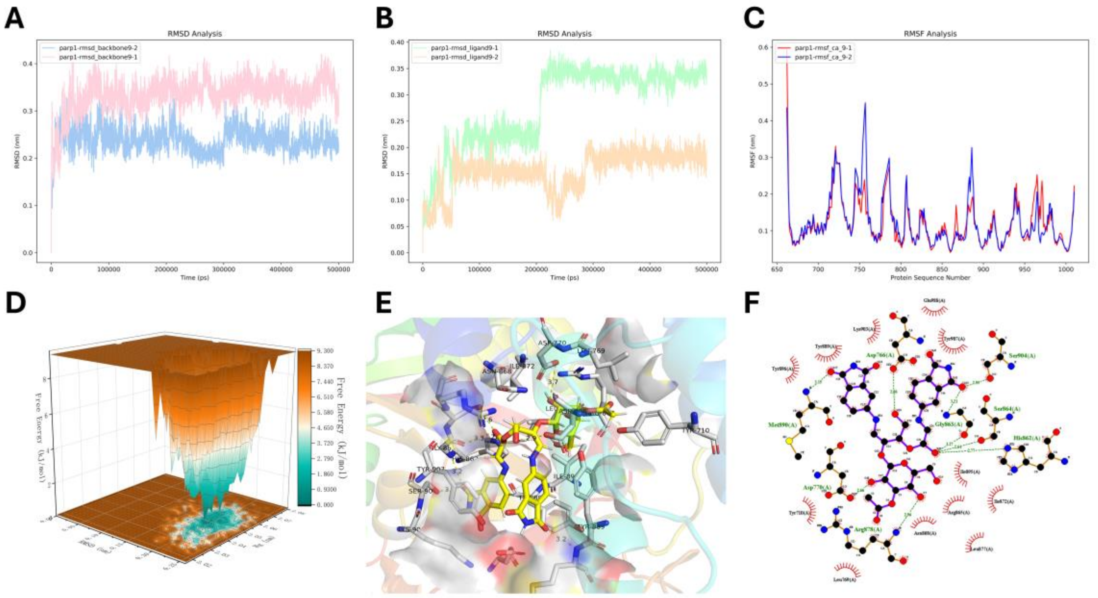

Figure S6. Comprehensive Analysis of compound 9 and PARP-1 Interactions. (A) RMSD values for PARP-1. (B) RMSD values for compound 9. (C) RMSF of the C-alpha of compound 1. (D) Gibbs Free Energy landscape of compound 9/PARP-1. (E) Three-dimensional visualization of the PARP-1-compound 9 interaction. (F) Two-dimensional representation of the PARP-1-compound 9 interaction.
